# Physiological Signal-Based Method for Measurement of Pain Intensity

**DOI:** 10.3389/fnins.2017.00279

**Published:** 2017-05-26

**Authors:** Yaqi Chu, Xingang Zhao, Jianda Han, Yang Su

**Affiliations:** ^1^State Key Laboratory of Robotics, Shenyang Institute of Automation, Chinese Academy of SciencesShenyang, China; ^2^Shengjing Hospital of China Medical UniversityShenyang, China

**Keywords:** feature extraction, feature selection and reduction, pain intensity quantification, physiological signals, pattern classification

## Abstract

The standard method for prediction of the absence and presence of pain has long been self-report. However, for patients with major cognitive or communicative impairments, it would be better if clinicians could quantify pain without having to rely on the patient's self-description. Here, we present a newly pain intensity measurement method based on multiple physiological signals, including blood volume pulse (BVP), electrocardiogram (ECG), and skin conductance level (SCL), all of which are induced by external electrical stimulation. The proposed pain prediction system consists of signal acquisition and preprocessing, feature extraction, feature selection and feature reduction, and three types of pattern classifiers. Feature extraction phase is devised to extract pain-related characteristics from short-segment signals. A hybrid procedure of genetic algorithm-based feature selection and principal component analysis-based feature reduction was established to obtain high-quality features combination with significant discriminatory information. Three types of classification algorithms—linear discriminant analysis, *k*-nearest neighbor algorithm, and support vector machine—are adopted during various scenarios, including multi-signal scenario, multi-subject and between-subject scenario, and multi-day scenario. The classifiers gave correct classification ratios much higher than chance probability, with the overall average accuracy of 75% above for four pain intensity. Our experimental results demonstrate that the proposed method can provide an objective and quantitative evaluation of pain intensity. The method might be used to develop a wearable device that is suitable for daily use in clinical settings.

## Introduction

From an evolutionary angle of vision, pain is considered as an unpleasant emotional and sensory experience that may be associated with a real or potential tissue damage (Hudspith et al., 2006; Loeser and Treede, 2008). Nowadays, pain is one of the most significant clinical symptoms that can be utilized to detect the acuteness and degree of a patient's injury. Moreover, pain is considered as a warning to danger, and often indicates the site of a lesion; its intensity serves as an indicator of patient well-being. A precise prediction of pain intensity could provide valuable insights in situations in which it can be utilized effectively to ultimately determine the position of pain and accordingly to formulate a reasonable therapeutic schedule. Therefore, pain prediction could enhance the quality of daily life for patients in the health-related field of rehabilitation, in-home healthcare and medical emergency services.

As a subjective first-person experience (Loeser and Treede, 2008), pain does not only reflect perceptual status but would also be substantially affected by psycho-physiological conditions (e.g., fluctuations in attention or alertness) and even psychosocial contexts (e.g., the age or gender of individuals) (Aslaksen et al., 2007). In clinical practice, self-description is the gold standard approach for the determination of the absence, presence, and intensity of pain (Cruccu et al., 2010; Haanpää et al., 2011), such as Numeric Pain Rating Scales (NPRS), Verbal Rating Scales (VRS), and Visual Analog Scales (VAS) (Frampton and Hughes-Webb, 2011). These self-reported scales are especially well applied and validated in cancer patients (Caraceni et al., 2002). In addition, the McGill Pain Questionnaire (MPQ) and Brief Pain Inventory are also used to assess the wider pain perception in multidimensional scales (Frampton and Hughes-Webb, 2011). While self-descripted pain provides important clinical reference indicators and proves to be a valid method for the adequate therapy of patients suffered from pain in most situations (Brown et al., 2011), it would fail to be applied in certain vulnerable populations. Individuals with communicative impairments or disturbance of consciousness, including older adults with dementia and intensive care unit patients in vegetative state, coma, and minimally conscious state (Herr et al., 2006; Schnakers and Zasler, 2007), may not be able to provide effective and credible self-reports of pain (Li et al., 2008). For those populations, lack or any inaccuracy indicators used to evaluate pain may lead to suboptimal or inappropriate treatment of pain, which may bring about various additional clinical issues, such as the deterioration of chronic pain and psychological distress or depression (Roulin and Ramelet, 2012). Furthermore, the self-reported pain is very subjective and unable to be obtained in real-time.

Recent developments in objective pain assessment have concentrated mainly on recognition and prediction from human behaviors, such as vocalizations (Puntillo et al., 2004), body motions (Young et al., 2006), and facial expressions (Lucey et al., 2011; Kaltwang et al., 2012; Irani et al., 2015). While behavioral methods exist, they also may be inapplicable in individuals with paralysis or other motor disorders affecting behaviors. By observing the face of an individual, a huge number of features related with affective state can be extracted, including pain state. However, facial expression-based pain recognition need track the special facial regions of the users, which can be very cumbersome and tedious in the clinical application. Meanwhile, researchers' effort has been shifted to target toward a physiology-driven pain prediction that does not rely on individual's facial or volitional behaviors (Shankar et al., 2009; Treister et al., 2012). Those studies have focused on diverse bio-physiological signals, such as heart rate variability (De Jonckheere et al., 2010, 2012; Faye et al., 2010; Logier et al., 2010), skin conductance or electro-dermal activity (Harrison et al., 2006; Treister et al., 2012), electromyography (Oliveira et al., 2012), electroencephalography (Nir et al., 2010; Huang et al., 2013), and functional magnetic resonance imaging (fMRI) (Marquand et al., 2010; Brown et al., 2011). Recently, pain assessment method implemented by multi-modality signals has been confirmed to be highly effective, some even outperforming single-signal mode markedly (Werner et al., 2014; Kächele et al., 2015). However, the most physiology-driven pain researches just provide statistically significant correlations between several bio-physiologic signals and the presence of pain. They are qualitative assessments for discriminating the presence or absence of pain. Few measurements can predict the intensity of pain as a level indicator. Hence, despite many remarkable researches, there is no acceptable pain assessment method based on physiological signals of human.

With the increasing availability of wearable smart devices, many researchers focus on developing a non-invasive integrated system for health monitoring. An appropriate interpretation of the signals recorded by wearable devices equipped with sensors can help monitor, evaluate and eventually solve health problems related pain. In this article, the focused goal is the quantitative measurement of pain intensity from multi-physiological signals obtained by wearable sensors in the customer market. At present, only few studies have been carried out the procedure of automatic recognition of pain intensity from physiological signals. Olugbade et al. applied electromyography (EMG) and body motions in combination with Support Vector Machines (SVM) and Random Forests (RF) as classifiers to recognize three pain intensity (Olugbade et al., 2015). Kachele et al. used EMG, skin conductance level (SCL) and electrocardiogram (ECG) incorporated with unsupervised and semi-supervised learning to establish a personalized system of continuous pain intensity recognition (Kachele et al., 2016). However, these assessment procedures are quite complicated and time-consuming, especially feature extraction. Moreover, the latter focuses on a personalization scenario where the recognition process may not be widely used in the general population.

In this paper, we proposed a convenient and objective method of pain intensity recognition based on multiple physiological signals. The technique uses a hybrid of genetic algorithm (GA) with principal component analysis (PCA) to obtain optimized feature combination related with pain states. And three kinds of classification methods are compared to establish more appropriate recognition models. The presented system that complements the self-reported pain can thus be adapted to general persons.

The remainder of this paper is structured as follows. In Section Dataset and Experimental Protocol, we provide a short presentation of physiological signals used in pain recognition and experiment of pain induced by electrical stimulation. Section Feature Processing elaborates a thorough feature processing phase, including feature extraction, feature selection, and feature reduction. In Section Pattern Classification, we present the three classifiers for pain intensity recognition system. Thorough experimental results and discussion are described in Section Experimental Results and Discussion. Section Conclusions and Outlook provides our findings and ideas for future study in this field.

## Dataset and experimental protocol

### Subjects

Six subjects (4 males and 2 females, numbered 1–6) aged 22–25 years (standard deviation [SD] = 3.0) were recruited in this study. All participants were healthy, without any history of medical illness, neurological or psychopathological disorders, and none had a history of chronic pain. All the experimental protocols of this study were approved by the ethics committee of China Medical University. Moreover, all subjects were fully informed about the procedures, risk, and benefits of the study, and written informed consent was obtained from all subjects before the study.

The algorithm design and performance validation in this pain intensity measurement were implemented for the databases constructed from physiological signals of each subject. The signals were obtained with the procedure described in Section Physiological Signals and Preprocessing and Pain Induction Protocol. Moreover, we continuously collected 1-week data from the same participant, thus generating a 7-day dataset. For the database of each subject, we randomly chosen 75% of the samples as the training set, and the rest as the testing set. For the 7-day database, data from six randomly chosen days were used as the training set, and the rest were used as the testing set (leave-one-out method).

### Physiological signals and preprocessing

Acquisition of high-quality physiological data is of utmost importance for the pain intensity recognition system. The selection of available physiological signals that are used as input is the first consideration in the pain detection system. It is expected that the physiological signals reflect the effect of pain on the activity level of the nervous system. However, unlike self-reporting or facial recognition of pain, where the truth class labels of pain intensity for a given sample are self-evident, a high-confidence physiological signal in an underlying pain intensity is not easy to be acquired. Furthermore, it is difficult to determine whether the variability of the physiological signals is specific to pain or whether they represent a general response to factors such as thought, cognition, emotion and environment.

Since we were decided to exploit a practical quantitative and convenient system, there was a limitation on the selection of available signals. Though facial electromyograms and electroencephalograms would be expected to be useful, the attachment of electrodes to the face and scalp is complicated and not suitable for practical application. In our study, the selected physiological signals were blood volume pulse (BVP), electrocardiogram (ECG), and skin conductance level (SCL). These signals reflect the activity level of the autonomic nervous system, which is connected with the secretory activity of cardiac muscles and internal organs. As a wearable device, we use the Infiniti 3000A platform developed by Thought Technology Ltd., Quebec, Canada. The Infiniti 3000A apparatus is a compact sensing platform with 10 isolated channels for recording signals. It is small enough to attach to a portable computer and integrates to commercial sensors via custom cabling. The sampling frequency was fixed at 256 Hz for all the three channels.

**Figure 1:**
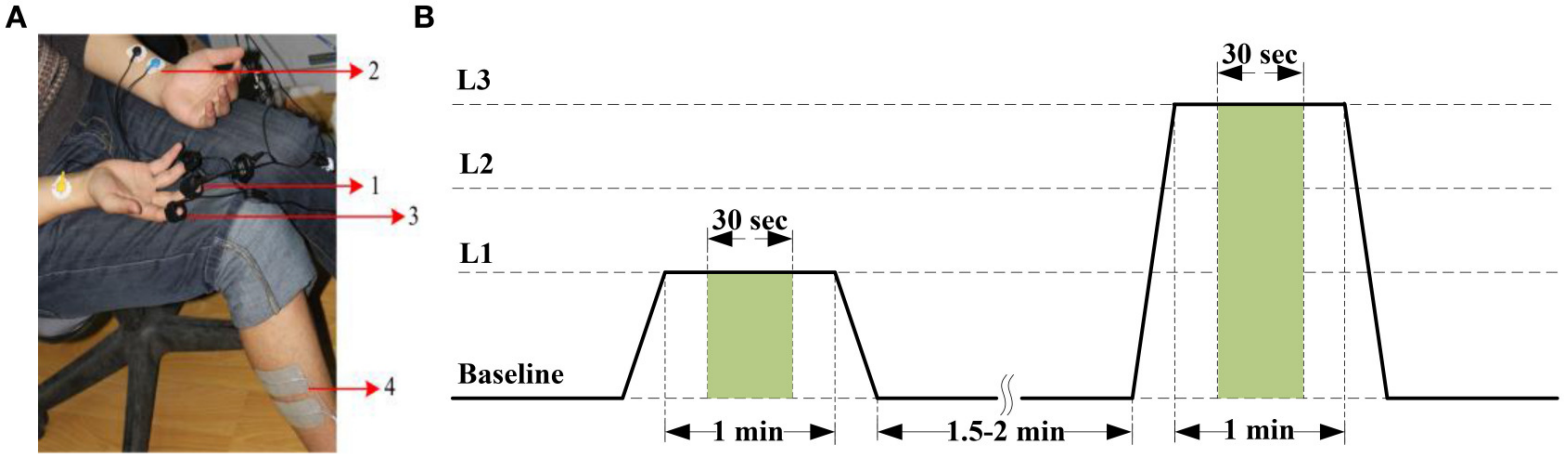
**(A)** Pain recording scenario. Positioning of sensors and electrical stimulation electrodes: (1) BVP-Flex/Pro sensor, (2) EKG-Flex/Pro sensor, (3) SC-Flex/Pro sensor, and (4) Electrical stimulation electrodes. **(B)** Pain induction protocol. The stimulus levels are represented by current intensity L1 to L3. The features are extracted from the green window of length 30 s.

The BVP signal is derived from a photoplethysmographic (PPG) sensor that monitors blood volume in capillaries and arteries by emitting an infrared light through the tissues. Vasomotor activity, which controls blood vessel diameter, is regulated by the sympathetic nervous system (Babchenko et al., 2001). Hence, changes in BVP amplitude reflect instantaneous sympathetic activation. Most PPG sensors can be placed anywhere on the body, with the finger as the most common location for recording a BVP signal. In this study, A BVP-Flex/Pro sensor was placed against the palmar surface of the middle finger of the right hand with an elastic strap or a short strip of adhesive tape to acquire BVP signals (Figure 1A). In the preprocessing phase, a 4-order Butterworth bandpass filter with gain 3, cutoff frequencies [30, 200] Hz was applied to eliminate the bursts in the BVP signal.

The ECG, an electro-physiological signal that is associated with the electrical activity of the sinuatrial node, reflects the cardiovascular activity. Additionally, ECG responses to external stimuli (such as pain stimuli and stress) can produce large variability in a given subject's physiological signal (Sriram et al., 2009). Therefore, we can employ ECG signal to extract universal information about pain state or intensity. The ECG signal was measured from both surface of upper arms with the two-electrode method based on lead I in our experiment (Figure 1A). For the preprocessing of the ECG trace collected by using non-invasive surface electrodes, a typical high-pass and moving-average filter (Milanesi et al., 2008; Sidek et al., 2014) were used to remove a low frequency baseline drift due to electrode contact noise, respiratory effects, and motion artifacts. Otherwise, we employed an adaptive linear interpolation algorithm to perform baseline correction before feature extraction.

The SCL is another signal that can easily be acquired from the body surface. It reveals variance in the electrical properties of the skin due to the secretory activity of the perspiratory glands. As it is directly regulated by the sympathetic nervous system, it is a good indicator of psychological or physiological arousal level due to external stimuli. Changes in the SCL signal induced by a painful stimulus were validated to be used as a tool to monitor nociceptive stimulation and pain (Harrison et al., 2006; Storm, 2008). The SCL signal was traced from two Ag/AgCl electrodes attached to the tip of the index and ring fingers on the palm-side of the right hand in this study (Figure 1A). In the preprocessing phase, a moving average smoothing was carried out to subtract artifacts. Furthermore, the raw SCL data was down-sampled by a factor of 1/2 to reduce the computational complexity in feature extraction phase.

### Pain induction protocol

As in many pain researches, there are variety ways that can be used to induce pain, such as thermal or cold pain stimulation (Appelhans and Luecken, 2008; Kachele et al., 2016), mechanical pain elicitation (Matsunaga et al., 2005; Shankar et al., 2009), and electrical shock (Oliveira et al., 2012; Zhang et al., 2016). In our study, pain was induced by an electrical stimulator (MotionStim8; Medel GmbH, Hamburg, Germany), which can generate a current square wave with a certain pulse width. The amplitude and frequency of the current are adjustable. The value of the pulse amplitude can be used as an objective index of pain intensity. In the experiment, the frequency of MotionStim8 was set to 2 Hz. In order to avoid interference of the stimulator with the sensors, especially the ECG sensor, the stimulation electrodes were placed on the tibialis anterior muscle of the right leg, as far away as possible from the sensors (Figure 1A).

**Figure 2:**
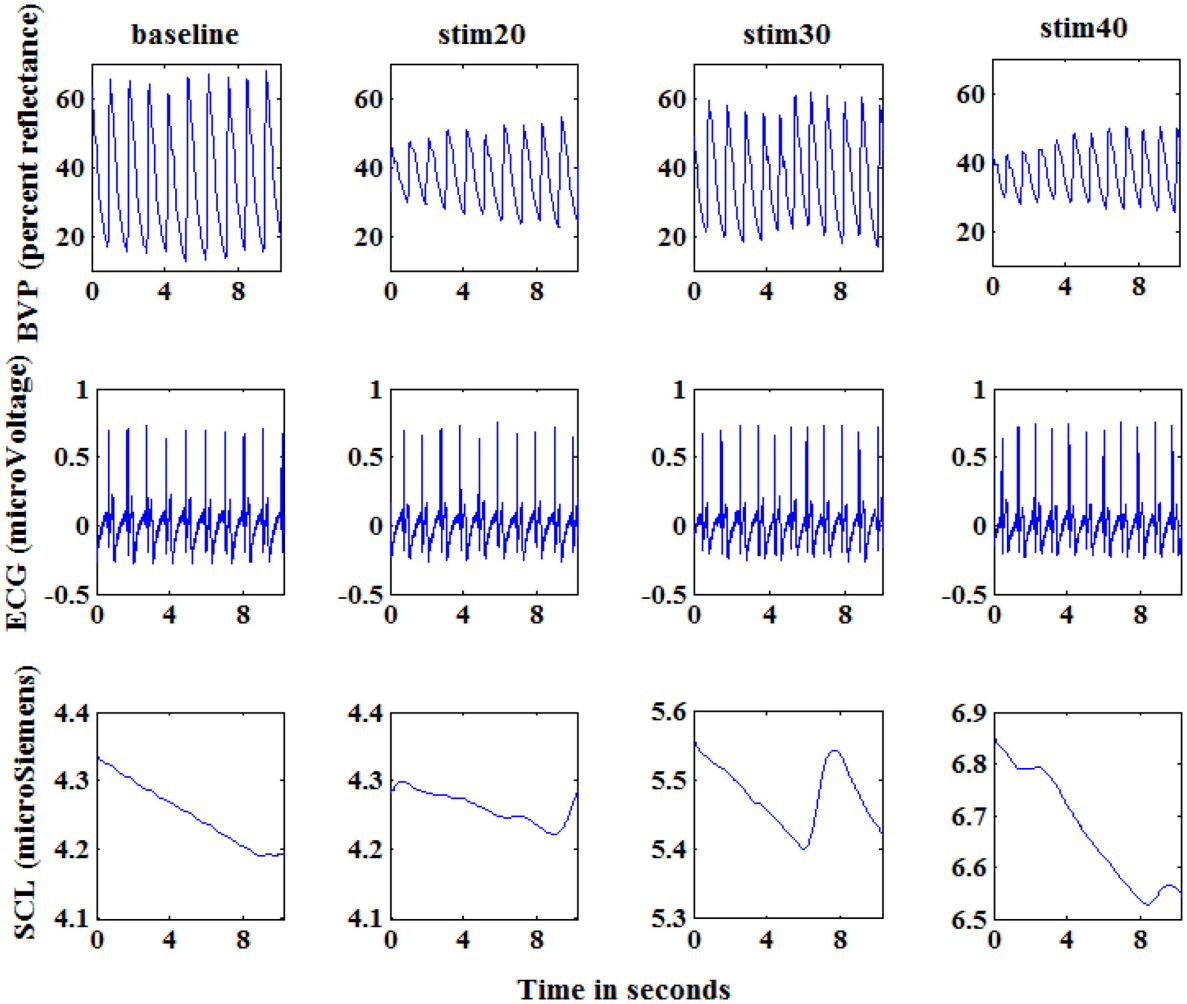
**Physiological signals of a subject at baseline, stim20, stim30, and stim40**. From top to bottom: blood volume pressure (BVP; percent reflectance), electrocardiogram (ECG; microVoltage), and skin conductance (SCL; microSiemens). Each graph shows 10 s of response. The segments shown here are visibly different for the four states.

Before stimulation phase, the current intensity was calibrated according to the subjects' self-reports. An electrical stimulus with 20 mA induced the sensation of pain was as pain starts (threshold). An electrical stimulus with 40 mA induced intense pain was as barely bearable pain (tolerance). Then, we divided the range between threshold and tolerance into 3 equally spaced intervals, 20-mA stimulus (stim20, L1), 30-mA stimulus (stim30, L2), and 40-mA stimulus (stim40, L3) with additional pre-stimulus (baseline, L0). During the stimulation experiment, each of the different current intensity was proceed for 1 min followed by a recovery period of 1.5–2 min (Figure 1B). To eliminate stimulation adaptability and time correlations, the sequence of stimuli and duration of the recovery period were randomly designed. Pain elicitation was executed 30 sessions for each of the 3 calibrated intensities (L1–L3) in the same day. With the addition of baseline phase, a total number of 120 stimulation trials were obtained for each subject. Each session took approximately 15 min. To minimize motion artifacts, the participants were requested to be as relaxed as possible during the stimulation phase. Moreover, the recording scenario that led to the multi-day dataset was continuously carried out 7 days for one person in the same condition. Figure 2 shows physiological traces from a participant in the four pain states.

## Feature processing

**Figure 3:**
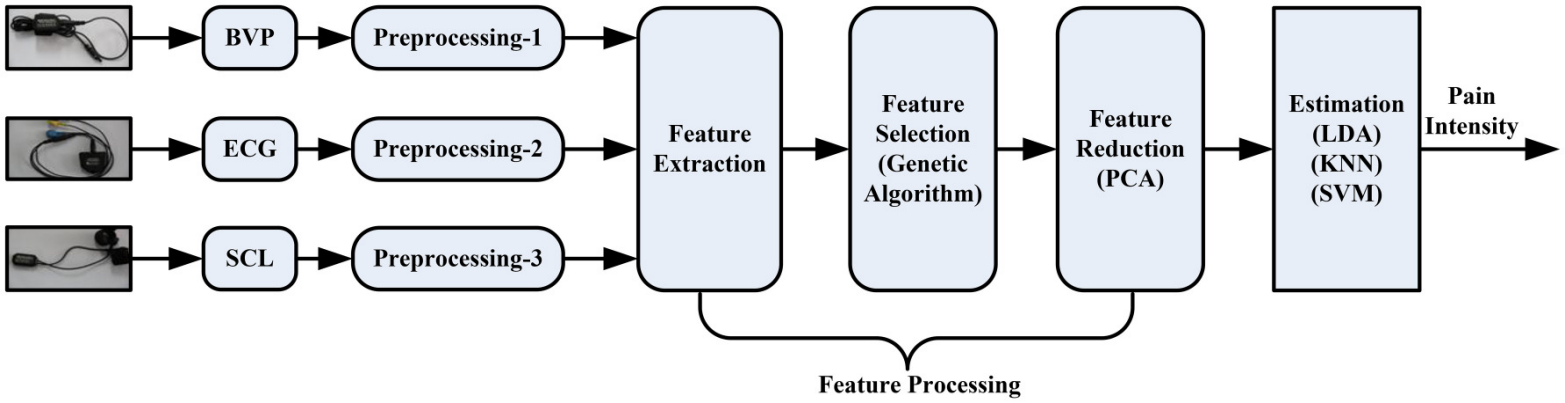
**Processing pipeline of the pain intensity recognition system**.

The overflow of the proposed pain intensity estimation system can be seen in Figure 3. The pipeline consists of raw signal input, independent preprocessing according to Section Physiological Signals and Preprocessing, feature processing, and finally pain intensity recognition. In this section, the proposed feature processing phase is introduced, including feature extraction, Section Feature Selection and Feature Reduction.

### Feature extraction

From the Figure 2, we can see that the amplitude of physiological signals obviously varied according to the level of electrical stimulus. Based on this observation, we gathered a variety of basic statistical features to evaluate the response to the pain-inducing stimuli, such as maximum, minimum, median values of data. Furthermore, these features can easily be calculated in an online way, which makes them more suitable for real-time recognition system of pain intensity.

The statistical features can be calculated for each of the signal channels as follows. Let the three preprocessed signals—the digitized BVP, ECG and SCL waveforms from any one of the four pain states segments—be designated by *X*^*i*^, *i* = 1, 2, 3 respectively. Let $X_{n}^{i}$ represent the value of the *n*^*th*^ sample of the *i*^*th*^ signal, where *n* = 1, 2, …, *N* with *N* in range of 3,840 to 7,680. Let ${\tilde{X}}_{n}^{i}$ refer to the normalized signal (zero mean, unit variance), formulated as:

(1) $$\begin{array}{r} {\tilde{X}}_{n}^{i}=\frac{X_{n}^{i}-\mu^{i}}{\sigma^{i}}\;i=1,2,3 \end{array}$$

where μ^*i*^ and σ^*i*^ are the means and standard deviations of *X*^*i*^ as presented below. Let $f_{j}^{i}$ represent the *j*^*th*^ features of the *i*^*th*^ signal.

• The means of the signals: $f_{1}^{i}$:

(2) $$\begin{array}{r} \mu^{i}=\frac{1}{N}\overset{N}{\underset{n=1}{\sum }}X_{n}^{i}\;i=1,2,3 \end{array}$$

• The standard deviations of the signals: $f_{2}^{i}$:

(3) $$\begin{array}{r} \sigma^{i}={\left(\frac{1}{N-1}\overset{N}{\underset{n=1}{\sum }}{\left(X_{n}^{i}-\mu^{i}\right)}^{2}\right)}^{1/2}\;i=1,2,3 \end{array}$$

• The means of the absolute values of the first differences of the signals: $f_{3}^{i}$:

(4) $$\begin{array}{r} \theta_{1}^{i}=\frac{1}{N-1}\overset{N-1}{\underset{n=1}{\sum }}|X_{n+1}^{i}-X_{n}^{i}|\;i=1,2,3 \end{array}$$

• The means of the absolute values of the first differences of the normalized signals: $f_{4}^{i}$:

(5) $$\begin{array}{r} {\tilde{\theta }}_{1}^{i}=\frac{1}{N-1}\overset{N-1}{\underset{n=1}{\sum }}|{\tilde{X}}_{n+1}^{i}-{\tilde{X}}_{n}^{i}|=\frac{\theta_{1}^{i}}{\sigma^{i}}\;i=1,2,3 \end{array}$$

• The means of the absolute values of the second differences of the signals: $f_{5}^{i}$:

(6) $$\begin{array}{r} \theta_{2}^{i}=\frac{1}{N-2}\overset{N-2}{\underset{n=1}{\sum }}|X_{n+2}^{i}-X_{n}^{i}|\;i=1,2,3 \end{array}$$

• The means of the absolute values of the second differences of the normalized signals: $f_{6}^{i}$:

(7) $$\begin{array}{r} {\tilde{\theta }}_{2}^{i}=\frac{1}{N-2}\overset{N-2}{\underset{n=1}{\sum }}|{\tilde{X}}_{n+2}^{i}-{\tilde{X}}_{n}^{i}|=\frac{\theta_{2}^{i}}{\sigma^{i}}\;i=1,2,3 \end{array}$$

• The minimum: $f_{7}^{i}$:

(8) $$\begin{array}{r} Min=minimum\left(X_{n}^{i}\right)\;i=1,2,3 \end{array}$$

• The maximum: $f_{8}^{i}$:

(9) $$\begin{array}{r} Max=maximum\left(X_{n}^{i}\right)\;i=1,2,3 \end{array}$$

• The minimum ratio: $f_{9}^{i}$:

(10) $$\begin{array}{r} minRatio\;=\;\frac{Min}{N} \end{array}$$

Here, *N* represents the length of the signal.

• The maximum ratio: $f_{10}^{i}$:

(11) $$\begin{array}{r} maxRatio\;=\;\frac{Max}{N} \end{array}$$

Here, *N* represents the length of the signal.

• The range of the signal: $f_{11}^{i}$:

(12) $$\begin{array}{r} range=Max-Min \end{array}$$

Besides, we also calculated the median value of the signal as $f_{12}^{i}$. Hence, each channel was characterized by 12 features. Considering the time lag of pain reaction, we selected 30 s signal for feature analysis in the 1-min data. For obtaining the subtle changes of the signals in a trial, we segmented the 30 s signal by a sliding window with 3 s sliding step (the window length was 3 s) to obtain the feature windows. Hence, a sample dataset of 40 × 36 can be obtained for each experiment session consisted of 4 trials. For single subject with 30 sessions, the size of sample dataset was 1,200 with 36 feature dimensions.

The BVP, ECG, and SCL are dependent on each participant's initial physiological level. Even when these signals are measured from the same person, they are likely day-dependent due to variations in mental state affected by emotion, variations in physiology caused by sleep or diet, or variations in the sensor's connectivity with skin (Sun et al., 2010). To eliminate the intra-subject and day-to-day variation factor, a normalization stage for each feature was applied in feature vector set *F*. Refer to (13), the first was to subtract the minimum value from each feature. Then, the features are divided by the range to make all the values lie between 0 and 1.

(13) $$\begin{array}{r} F{\left(m\right)}_{norm}=\frac{F\left(m\right)-V_{min}\left(m\right)}{V_{max}\left(m\right)-V_{min}\left(m\right)} \end{array}$$

where, *V*_*min*_(*m*) = *min*{*F*_*n*_(*m*)}, *V*_*max*_(*m*) = *max*{*F*_*n*_(*m*)}, ∀*n* ∈ |*F*|.

### Feature selection and reduction

Note that not all of these features are independent. Some features are a linear combination of other features (e.g., ${\tilde{\theta }}_{1}^{i}$ and ${\tilde{\theta }}_{2}^{i}$) or a non-linear property. To obtain high-quality features with significant discriminatory information, a hybrid combination of genetic algorithm (GA) and principal component analysis (PCA) was proposed. This combination was motivated by the roles of the two algorithms: GA selects from a feature set to remove useless and redundant features, while PCA linearly transforms a feature set. Since feature selection is non-linear, the hybrid of these two ways could provide a more powerful separation than either alone.

The GA is a biologically inspired stochastic algorithm based on some optimization criterion that mimics natural evolution. The GA is naturally applicable to feature selection since the problem has an exponential search space (Oh et al., 2004). Furthermore, the distinct specialty of this algorithm is that it always maintains a set of solutions (called chromosomes or individuals) in a population. To simulate the biological evolution, the GA selects fitter chromosomes at each generation by some genetic operations, such as crossover and mutation. The traditional procedures of steady-state GA are described below.

1. Initialize population *P*;
2. Repeat {
3.     select two parent chromosomes *p*_1_ and *p*_2_ from *P*;
4.     offspring = crossover (*p*_1_, *p*_2_);
5.     mutation (offspring);
6.     replace (*P*, offspring);
7.     fitness (offspring);
8. } until (stopping condition);

For the feature selection problem, a finite bit string with *d* binary digits is usually used to represent chromosomes. A binary digit represents a feature. A value of 1 or 0 in the chromosomes means that the corresponding feature is selected or removed, respectively. For example, a chromosome 10100010 means that the first, third and seventh features are selected. Since we wanted to improve recognition rate, the classification accuracy from the classifiers was directly used as the fitness function to evaluate a chromosome (Lin et al., 2015). Based on the initial fitness evaluation, the chromosome for the next generation was selected by a selection mechanism that ensures fitter chromosomes have a higher probability to survive. In our design, a traditional roulette wheel selection strategy was applied. We also used the standard two-point crossover operator to obtain the offspring and Gaussian mutation operations at probability on each of the offspring produced from crossover. The crossover rate and mutation rate were set to 0.8 and 0.01, respectively. Besides, the size of population and the maximum generation in GA were set to 100 and 30, respectively. These parameters are tunable to be suitable for a specific dataset to improve performance.

The principal component analysis (PCA) is a well-known statistics method of multi-variable analysis and used orthogonal transformation to convert a set of observations of possibly correlated variables into a set of values of linearly uncorrelated variables (Karamizadeh et al., 2013), also known as principal components. Moreover, PCA is a tool to reduce dimensionality by searching a linear projection matrix of the dataset to a feature space with lower dimensions while retaining most of the information. This linear projection matrix is a meaningful basis to filter out the noise and reveal hidden information in the original feature space. The key advantages of the PCA are the decreased requirements for memory and capacity, it low noise sensitivity, and increased efficiency in a lower dimension. By solving the covariance matrix, a series of eigenvalues with the corresponding eigenvectors are computed and rank-ordered descendingly by quantifying how “principal” each variance is. The *k* largest eigenvalues are selected and the corresponding eigenvectors (*k* principal components) are used to construct the linear projection matrix. Then, a feature space with *N* dimensions is transformed to a space of *k* dimensions by this reduction matrix. For a detailed description of the PCA algorithm about mathematical derivation, the reader is referred to Bro and Smilde (2014).

A hybrid GA with PCA method is proposed, evaluated and implemented for comparison. As mentioned above, the GA algorithm provides a subset of features selected from raw features. Instead of feeding the PCA algorithm with all possible features, we used the selected subset of features as the input to the PCA. Note that the PCA algorithm is applied here for transforming features into a linearly uncorrelated space.

## Pattern classification

The features undergo processing were feeding to classifiers to identify a subject' pain intensity. We used three types of learning methods to train and investigate the performance of classifiers, including linear discriminant analysis (LDA), *k*-nearest neighbor (KNN) algorithm, and support vector machine (SVM). We evaluated classification performance for single-signal datasets and multi-signal datasets, as well as for multi-subject datasets and multi-day datasets.

The LDA is a commonly used statistical method that uses a linear discriminant function to classify a new data points. In the calculation process of the algorithm, a hyperplane is constructed by finding a linear combination of features to separate or characterize two classes of objects or events. The optimal linear combination or projection in a classical LDA is constructed by minimizing the within-class distance and maximizing the between-class distance simultaneously, thereby achieving maximum class separation (Yan et al., 2014). Since a LDA algorithm is a linear binary classifier, it is not suitable in multi-class situations. In our pain study, the classes are three pain intensity levels and the baseline state. Thus, we need a multi-task method to inference pain states. A one-vs.-rest strategy was used to address multi-class problems. For each class *l*, an independent LDA classifier was trained to separate data of class *l* and the rest class. In this paper, four trained LDA classifiers were applied in the test set. For a test sample, the recognition result was determined by selecting the class with the highest output value. Moreover, the classification accuracy of LDA was used as the fitness in the GA.

The KNN is a simple multi-class technique that operates by finding the *k* objects in the training set which are closest to the test sample by some metrics, such as measures based on distance (Weinberger et al., 2005). In order to classify an instance of the test set into a class, KNN calculates the distance between each instance of the training set and this test sample. Then, a prediction is determined as the most common class among this set of nearest neighbors, with each neighbor's vote being assigned a weight inversely proportional to that neighbor's distance from the test sample. The performance of a KNN classifier is primarily determined by the choice of *k* as well as the distance metric applied (Xiao and Chaovalitwongse, 2016). By effectively using prior knowledge such as distribution of the features, Euclidean distances were applied as the distance metric in our KNN classifier. In addition, the configurable parameter *k* of the *k*-nearest neighbor algorithm was set to 3 by multiple testing.

The SVM is a supervised learning algorithm by finding a high-dimensional discriminant hyperplane to separate observations into two class with a maximum margin, generally a soft margin (Vapnik, 2005). A regularization parameter was applied in the soft margin to penalize misclassification. By using the “kernel trick” to map the testing data points into a higher dimensional space, a support vector machine can construct an optimized separation hyperplane and make the mapped data easier to be separated. For pain recognition, randomization was executed using a computer-based list randomizer to construct the training data set for each subject. The preferential kernel function was the Gaussian or Radial Basis Function (RBF) kernel in this study. The SVM model was trained by cross-validation method with a grid search to find the optimal parameters of regularization and kernel. In this study, the SVM is optimal with a regularization of 5 and a Radial Basis Function kernel with width of 2.58.

In both of the KNN and SVM methods applied in pain recognition, the processed features were uniformed to zero mean and unit variance. Accordingly, the effect of feature variations in distance-based inference algorithms was reduced.

## Experimental results and discussion

### Initial results—single-signal analysis

**Figure 4:**
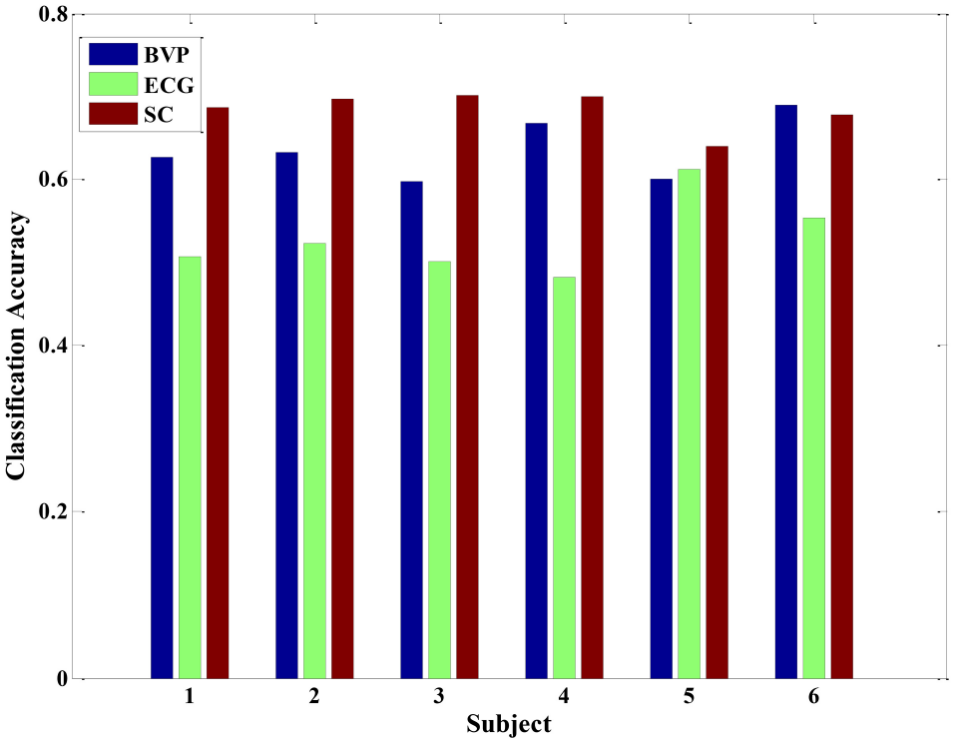
**The classification accuracy of the LDA algorithm for each physiological signal in each of the six subjects**.

Previous studies have performed qualitative analyses of pain based on a single physiological signal. For example, Harrison et al. revealed SCL as a promising indicator to predict the presence or absence of pain in hospitalized infants during painful and non-painful medical procedures (Harrison et al., 2006). However, in this study, we applied pattern recognition techniques to quantify pain intensity based on a set of physiological signals. Since there was not yet a priori knowledge of pain recognition, we originally established a quantitative model for pain intensity from individual's single physiological signal. For each signal, we extracted 12 statistical features to construct three types of feature set, including BVP-features, ECG-features, and SCL-features. The classification accuracy of the LDA algorithm was evaluated by using these three types of feature set. Figure 4 presents the recognition results gained with LDA algorithm for each physiological signal in each of the six study subjects.

From the Figure 4, the experimental results provide evidence to support our hypothesis that a single signal can be used to quantify pain intensity in some degree, with the average classification accuracy of 50% above. Furthermore, since the BVP and SCL signal were highly affected by the intensity of pain stimuli in our experiment, the classification accuracies were even higher than that of ECG signal alone. Especially, the average classification accuracy of SCL signal was 68.39% (±2.32%), and that of ECG signal was 53.01% (±3.40%). Unlike ECG, it can be observed that even the original SCL signal already presents an obvious variance with the pain stimulation in Figure 2. As the experimental results suggest, the extracted SCL features are a good indicator to identify the pain intensity. This is particularly suitable when it comes to integrate the proposed methods into wearable device. Since the SCL sensors are less intrusive and more convenient attached than BVP or ECG sensors, it can be easily embedded into wearable devices. Although the performance of LDA model for a single signal is considerable, the correct classification rate is relatively low. For improving the classification performance and obtaining better models, we considered applying multiple physiological signals with all-feature combination to quantify pain intensity. The idea was inspired to take full advantages of pain information contained in various signals.

### Results of the multi-signal model

**Figure 5:**
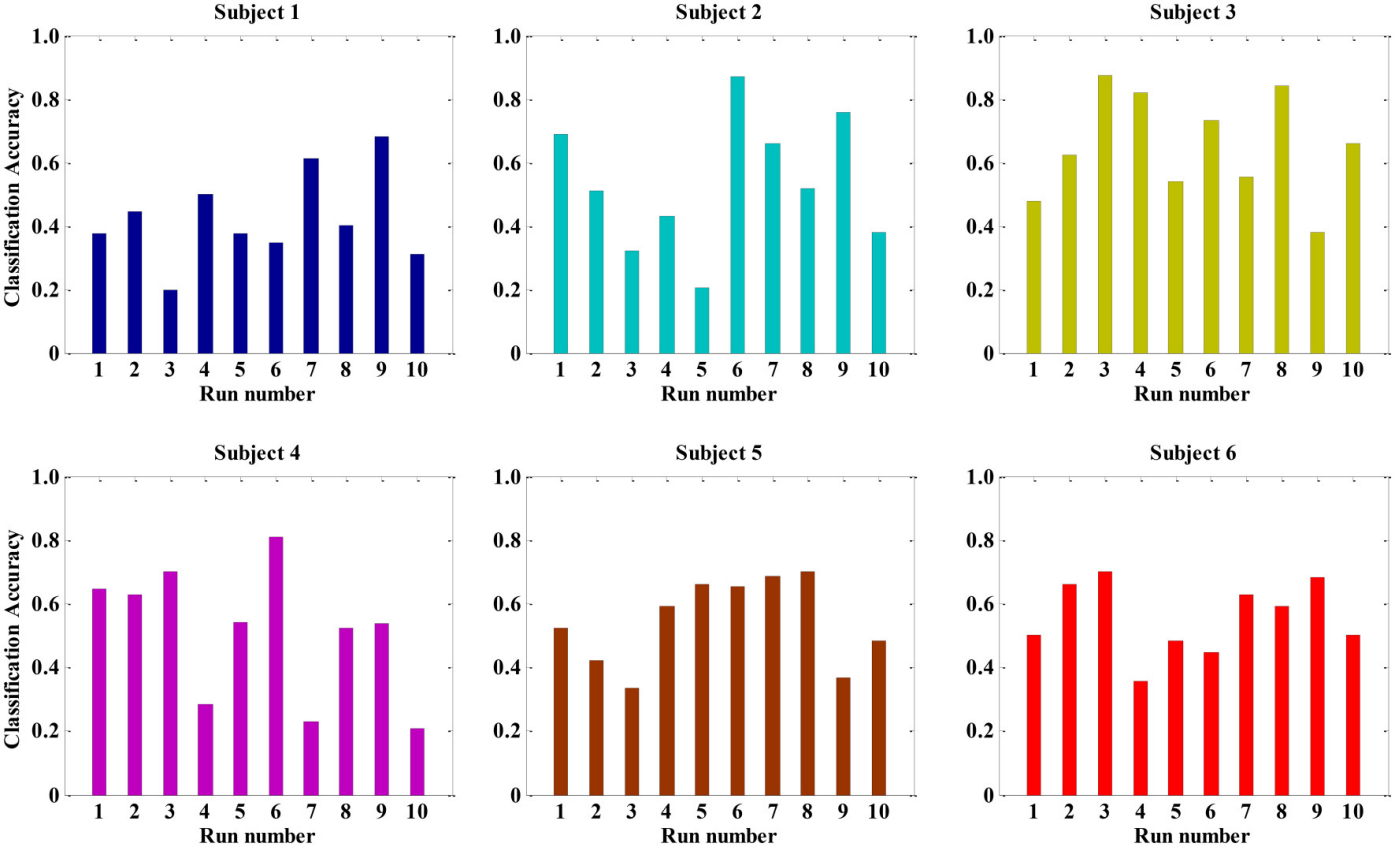
**The classification accuracy of running ten times LDA for all 36 features in each subject**.

Since we used multiple signals (BVP, ECG, and SCL), the total number of extracted features per sample was 36 (12 features per signal). We also selected the LDA algorithm as the classifier. The recognition accuracies of running 10 trials LDA are shown in Figure 5. The average accuracy of the LDA model was 42.49% (±14.31%) for subject 1, 53.78% (±20.84%) for subject 2, 65.12% (±16.57%) for subject 3, 51.07% (±20.52%) for subject 4, 54.30% (±13.62%) for subject 5, and 55.52% (±11.43%) for subject 6, respectively. The maximum classification accuracy of 10 trials LDA algorithm in the condition of multiple signals was approximately 90% (such as subjects 2 and 3). However, the variation of classification accuracy was large, ranging from 19.64% (subject 1) to 87.61% (subject 3). This finding indicated that the robustness of the multi-signal model established by LDA was insufficient. This may be because some extracted features are not independent and may be correlated. Furthermore, some features do not contribute or contribute little to the performance of the classification. A poor feature combination may greatly degrade the performance of the classifier. Hence, feature selection was needed to select a subset of good features.

In this study, we applied the genetic algorithm technique to seek and identify the potential informative features combinations, which contribute most to the performance of the classification. From other researches, it is obvious that the best accuracy of the LDA by using cross-validation method is considered as the indicator of the fitness function. Table 1 provide the experimental results of GA selection. The GA was run 15 times; each row lists the results from the corresponding run. In Table 1, the column “Best generation” indicates the generation number in which the optimal set of features was selected. And the average number of selected features was roughly 18. The performance of LDA was significantly improved with average accuracy of 72% above. In detail, the feature $f_{2}^{i}$, $f_{3}^{i}$, $f_{4}^{i}$, $f_{5}^{i}$, $f_{6}^{i}$, and $f_{7}^{i}$ computed from the three physiological signals were almost repeatedly selected by each running of the GA, yielding 18 features: μ^*i*^, σ^*i*^, $\theta_{1}^{i}$, ${\tilde{\theta }}_{1}^{i}$, $\theta_{2}^{i}$, ${\tilde{\theta }}_{2}^{i}$, *i* ∈ (*BVP, ECG, SCL*). It means that these features are the more robust feature combination than those that are rarely selected in the context of this pain experiment.

**Table 1:** **Experimental results of 15 runs of the GA**.

**Runs**	**Best generation**	**Number of features**	**Features selected**			**Best accuracy (%)**
			**BVP**	**ECG**	**SCL**	
1	20	16	100110100100	111011000010	011110000100	77.80
2	15	17	111011000010	001111000001	111011000001	84.20
3	10	17	011111000000	100110001000	111111100001	86.89
4	25	19	110111100100	011011000100	111011001100	79.95
5	17	20	111011000101	111011000001	111111001000	82.38
6	13	20	101101101000	011111001001	111011010010	75.38
7	19	18	110111000100	111011001100	101111000000	88.89
8	22	18	111011010010	010100100100	011111001001	84.28
9	18	19	011111001001	111100000100	111011100010	85.25
10	27	20	101111101101	010100110010	011011010010	72.30
11	19	17	111011100000	001111100001	111011000000	82.10
12	15	18	110111000100	100110001100	111100001110	75.35
13	22	17	111001000100	010111110000	011111100000	80.82
14	15	20	101110010001	111011110000	101111110000	79.08
15	25	20	011110001110	010111000110	110111100100	84.27

**Figure 6:**
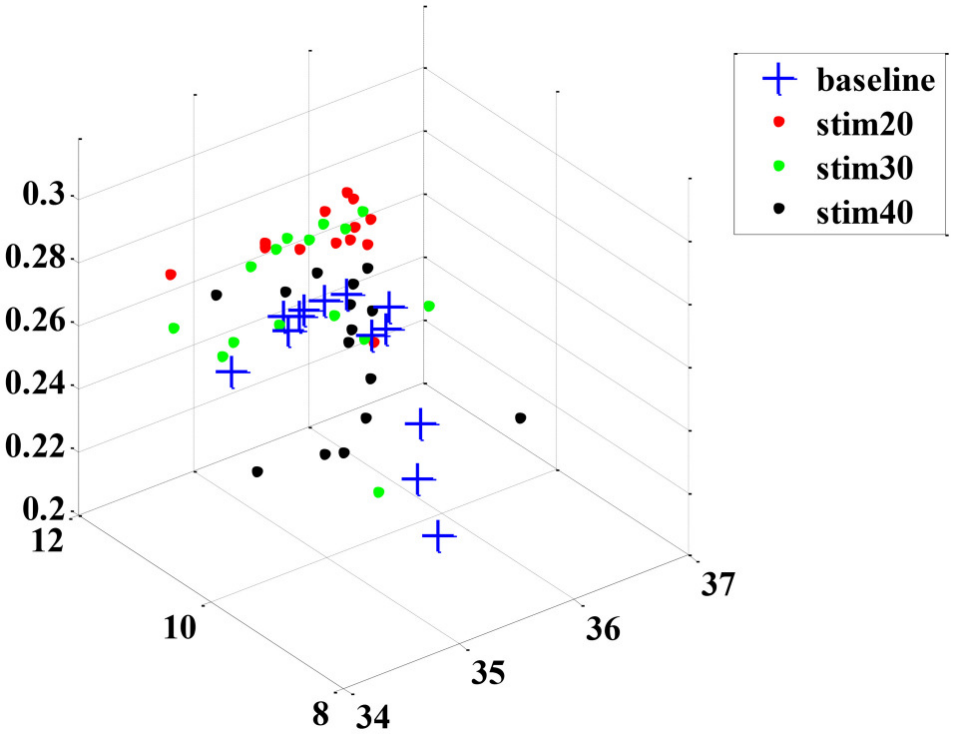
**An example sample set from subject 1 before dimension reduction projected onto the first three features**.

**Figure 7:**
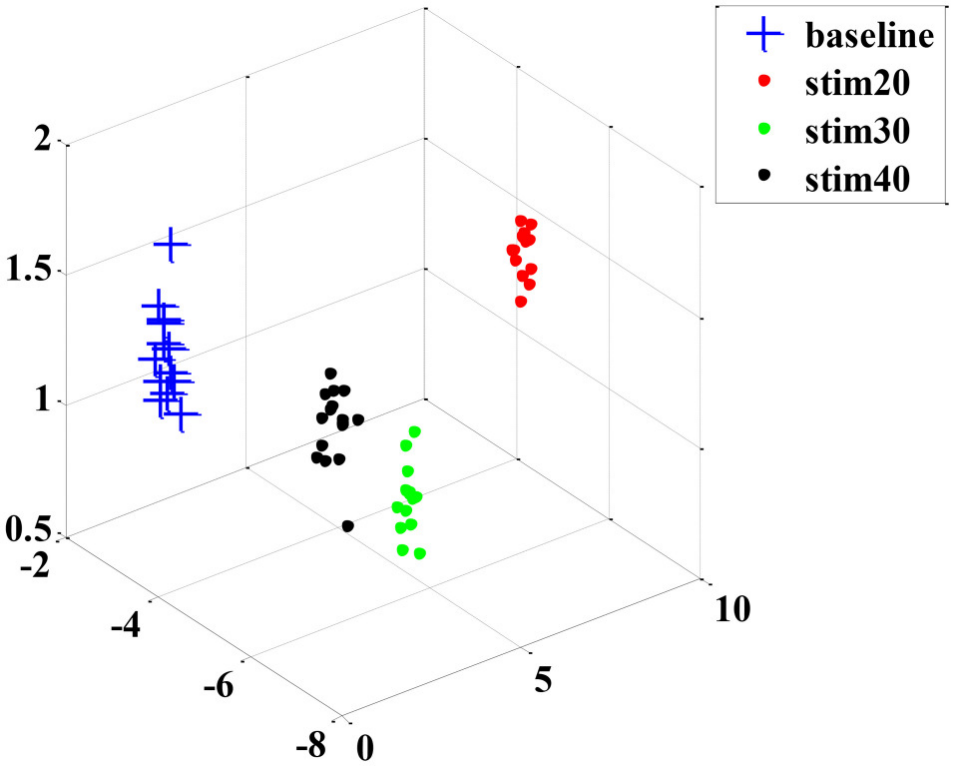
**An example sample set from subject 1 after dimension reduction projected onto the first three features**.

Although the trend of the feature combination proves their efficacy in distinguishing pain intensity, the 18 features selected by GA are not the best informative features for classification. This is because the genetic algorithm cannot easily eliminate the linear features. To solve this problem, we applied the feature reduction technique. Figure 6 shows samples of 18-dimension feature space projected onto the first three features from subject 1. The selected feature vectors from the same class aggregated a cluster with a large amount of variation, whereas the feature vectors from the different classes overlapped dramatically. Then, a PCA method was applied to reduce the redundant linear features. After data transformation, the feature vector samples within same class were clustered more compactly than did samples before transformation, and the feature vector samples from different classes are farther away from one another (see in Figure 7).

**Figure 8:**
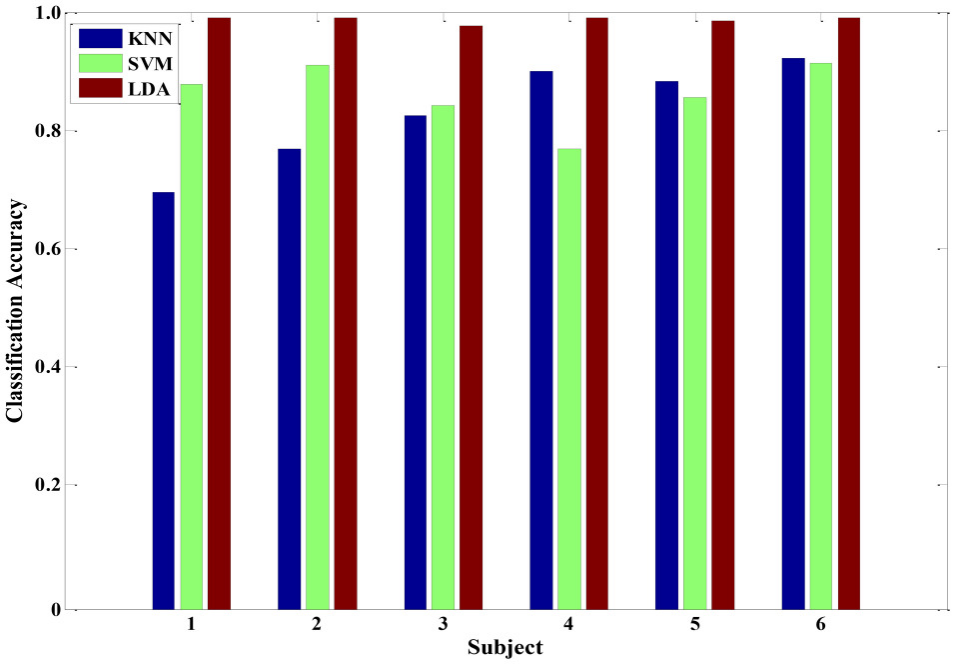
**The classification accuracy of three classifiers (KNN, SVM and LDA) after feature processing in each of the six subjects**.

After the feature processing stage by using the hybrid GA with PCA, the samples were fed to the LDA classifier to investigate the performance. In addition, we also tested the KNN and SVM algorithms for comparison. The corresponding recognition accuracy is presented in Figure 8. Using the optimized feature set, the average classification accuracy of three classifiers reached 80% or higher. Particularly, the performance of LDA algorithm was better than the other two algorithms, with 98% average accuracy. Those results prove that the proposed feature processing method is effective. And, the LDA classifier is more suitable in physiology-based pain intensity recognition for a single-subject scenario.

### Results of the multi-subject model

**Figure 9:**
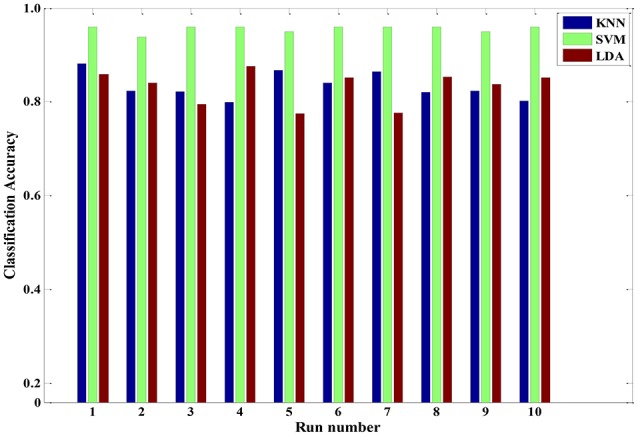
**The classification accuracy of running ten times three classifiers (KNN, SVM and LDA) for a multiple-subject scenario**.

In addition to discussing the recognition model of a single subject, we also analyzed samples constructed from multiple subjects. A sample set of multiple subjects was generated based on the six participants. Similarly, GA-based feature selection and PCA-based dimension reduction technique were carried out on the sample set comprising multiple subjects. The LDA, KNN, and SVM algorithms were implemented to train the corresponding models. Figure 9 shows the pain intensity recognition accuracy of running 10 times for the multi-subject model. The average categorize accuracy was 84.28, 83.94, and 96.47% for LDA, KNN and SVM, respectively. Overall, these three algorithms effectively identified pain intensity. The relevant multi-subject models established by LDA, KNN and SVM are feasible and appropriate. Besides, the multi-subjects model gained from SVM algorithm was better than the other two models. Every time we used the training SVM model to test the rest samples, the predicted accuracy was 92% or higher. Therefore, the trained SVM model can be used as a general model to quantify pain intensity for a multiple-subject scenario.

**Figure 10:**
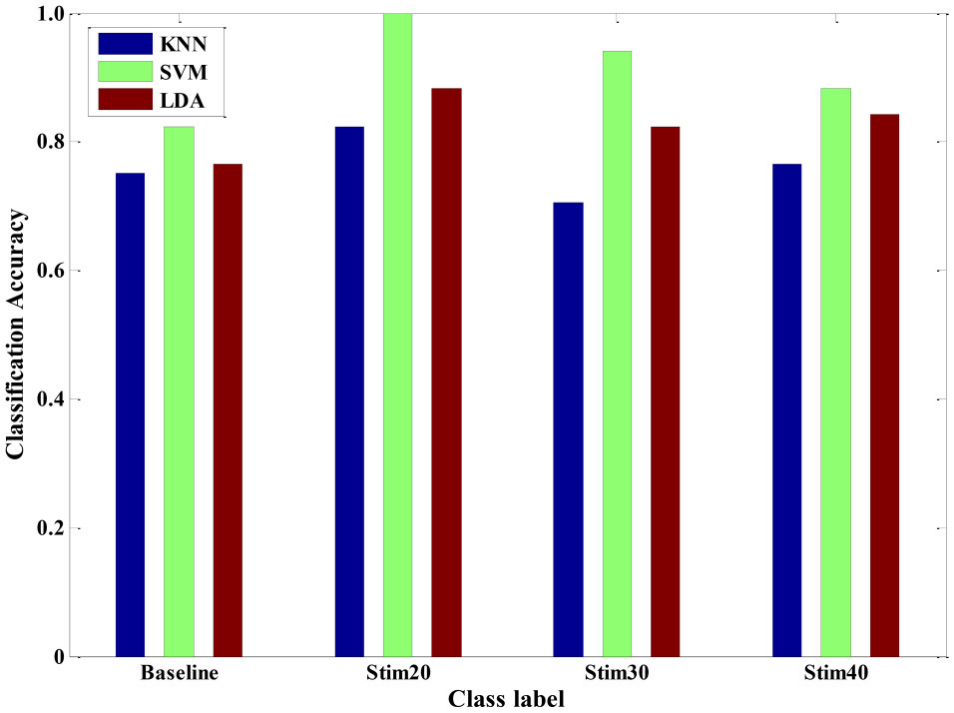
**Pain intensity recognition of three classifiers (KNN, SVM and LDA) for a between-subject scenario**.

Meanwhile, between-subjects' experimental validation was conducted by using the LDA, KNN, and SVM in a leave-one-subject-out cross-validation method, which is standard when dealing with multiple subjects. Here, the recognition modes were trained with dataset from randomly selected six subjects to predict the pain intensity of the remaining subject. It was repeated until all of the subjects had been part of the testing dataset. For a given between-subject scenario, we repeated 20 times and calculated the average accuracy for each pain intensity, respectively (see Figure 10). In detail, the average recognition accuracy of pain intensity with baseline, stim20, stim30, stim40 was 78.01, 90.10, 82.35, 82.90%, respectively. This is a more realistic condition in which the distribution of the training dataset and the testing dataset are dissimilar due to inter-individual variability. Moreover, the SVM algorithm was performed better than the KNN and LDA, with average accuracy of 91.18, 76.14, and 82.83%, respectively. These results demonstrate that the SVM classifier outperforms the other two classifiers for a between-subject scenario. Furthermore, the proposed system in the context of pain prediction would mitigate the inter-personal variance while preserving precise scalability.

### Results of the multi-day model

**Figure 11:**
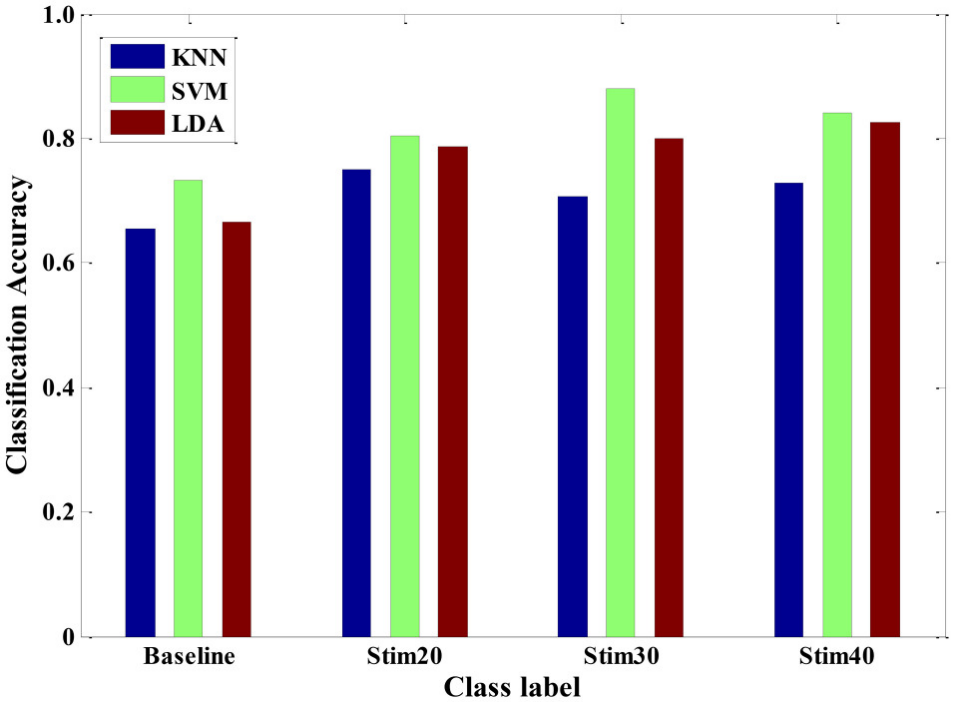
**Pain intensity recognition of three classifiers (KNN, SVM and LDA) for a multi-day scenario**.

Furthermore, we also conducted experimental evaluation by using the three types of classifiers in a multi-day scenario. For one person, we continuously carried out the pain induction experiment of 7 days and collected a seven-day dataset. A leave-one-day-out cross-validation method was applied to obtain the pain intensity of one person. The results of multi-day model are given in Figure 11. The average recognition rate was 71.02, 81.39, and 76.93% for KNN, SVM, and LDA, respectively. It implied that the trained SVM model seemed to be more suitable as a special model to quantify pain intensity of multiple days for one subject. In addition, the overall accuracy was roughly reduced from 87% of 1-day scenario to 70% of multi-day scenario. This may be mainly because that the signals measured from the same person are likely day-dependent. The quality of signals is affected by the intro-individual variance in mental state and environmental factor. However, our proposed pain intensity system is relatively fitted for practical applications of continuous and long-term pain monitoring.

## Conclusions and outlook

In this paper, we proposed a physiological signal-based method for convenient quantification of pain intensity induced by the external electrical stimulation. The proposed measurement provides a valid, physiology-based substitution of self-reported pain. The newly developed pain intensity measurement system consists of signal acquisition and preprocessing, feature extraction, GA-based feature selection and PCA-based feature reduction, and three types of pattern classifiers, including LDA, KNN, and SVM. Using the hybrid feature processing of GA with PCA, the training samples can be optimized to obtain a higher recognition accuracy while simultaneously reducing computational complexity as unrelated features are removed, such as maximum, minimum, median, et al.

In various experimental validations, our method was tested and the performance of three classifiers were evaluated, including single-signal analysis, multi-signal model, multi-subject scenario, and multi-day scenario. For a LDA model trained by a single signal, the SCL signal seems to provide a better result than that of the BVP or ECG signal. Moreover, after a series of feature process, the performance of LDA classifier was significantly improved for multi-signal model. And, the LDA classifier is more suitable in physiology-based pain intensity recognition for a single-subject scenario compared to the KNN and SVM. For the samples constructed from multiple subjects, the KNN, LDA, and SVM could categorize pain intensity with 83.94, 84.28, and 96.47% accuracy, respectively. We also compared the performance of the three classifiers for a between-subject scenario. It seems that the performance of SVM algorithm was slightly better. Therefore, the trained SVM model can be used as a general model to quantify pain intensity for other unseen population. Furthermore, the three classifiers could also reach high accuracy for a multi-day scenario. These findings suggest that the proposed pain intensity measurement method can be suitable for practical applications of continuous pain monitoring. From Figures 10, 11, we can see that the recognition accuracy of different pain intensities was different. Especially, the recognition rate at baseline was relatively low. This could be caused by the large amount of variation of physiological signals for various subjects and various days at baseline. There are numerous causes that may influence the physiological baseline signals, such as cognition, physical movement, and even physical or psychological status. These factors may also affect the model selection and even deteriorate the recognition ratio. In summary, our system was able to predict well above chance level between three levels of pain intensity with respect to previous researches (Olugbade et al., 2015; Kachele et al., 2016).

For our future work, an online recognition experiment of pain intensity based on multi-physiological signals will be conducted. Then, a system of continuously pain monitor will be developed. Eventually, we expect that the sensors of our proposed system would be executed as a wearable device that is suitable for daily use in clinical settings. In the case of online recognition, the input signal cannot be preprocessed and standardized in advance. A large amount of signal from previous offline experiments in various population must be collected before using to deal with the online prediction of unseen samples. Besides, the latency of the online system must be considered. The final prediction system is to provide a trade-off between lower latency and higher accuracy. Since the dataset generated in the current study was obtained by artificial electrical stimulation to induce different intensities of pain, this procedure is not appropriate for an online prediction system. Hence, more ways should be investigated in order to predict and quantify pain intensity perceived by a person. We furthermore plan to research various methods of feature selection to access to a comparison result.

## Author contributions

YC, XZ, JH, and YS conceived the conception and design of this research. YC executed the experiments, including acquisition and analysis of data for the work. YC, XZ, JH, and YS interpreted the experimental data. YC drafted the manuscript. XZ, JH, and YS revised the manuscript.

### Conflict of interest statement

The authors declare that the research was conducted in the absence of any commercial or financial relationships that could be construed as a potential conflict of interest.
